# Evaluations of exercise intolerance with cardiopulmonary exercise tests in an 18-year-old adolescent with pituitary stalk interruption syndrome: a case report

**DOI:** 10.1186/s12902-022-00986-9

**Published:** 2022-03-29

**Authors:** Wei Hung Sung, Shin Tsu Chang, Li Yun Teng, Ko Long Lin

**Affiliations:** 1 Department of Physical Medicine and Rehabilitation, Kaohsiung Veterans General Hospital, Kaohsiung, Taiwan; 2 Taoyuan General Hospital, Taoyuan, Taiwan; 3 Department of Physical Medicine and Rehabilitation, Tri-Service General Hospital, School of Medicine, National Defense Medical Center, Taipei, Taiwan

**Keywords:** Cardiopulmonary exercise test, Exercise intolerance, Pituitary stalk interruption syndrome, Growth hormone deficiency, Case report

## Abstract

**Background:**

Pituitary stalk interruption syndrome (PSIS) is a rare disease associated with different level of anterior pituitary hormone deficiency resulting with a variety of clinical manifestations which could limit exercise capacity. Cardiopulmonary exercise test (CPET) is valuable in differential diagnosis of exercise intolerance and exercise prescription.

**Case presentation:**

An 18-year-old male adolescent was diagnosed with PSIS at 4 years old, had undergone growth hormone supplement until puberty, and was referred to rehabilitation department due to exercise intolerance. We arranged pulmonary function test (PFT) and CPET to clarify the cause of limited capacity. The test result provided evidence of moderate functional impairment (54% of predicted maximal oxygen uptake) mainly affected by physical unfitness without significant cardiovascular or pulmonary limitations.

**Conclusion:**

CPET serves as a valuable tool for diagnostic purpose. Aerobic and resistance exercise training for the patient should be conducted promptly for better prognosis but under safe circumstances, with criteria which could be provided by CPET results.

## Background

Pituitary stalk interruption syndrome (PSIS) is a rare disease which often relates to anterior pituitary hormone deficiency with prevalence of 0.5 in 100,000 births [1–3]. Its distinct radiological manifestations in magnetic resonance imaging in aid to make the specific diagnosis included the triad of thin or absent pituitary stalk, hypoplastic anterior pituitary and ectopic posterior pituitary [4]. The etiology or pathophysiology has not yet well established, but some known gene mutations are considered to be contributive to hypoplasia of pituitary stalk and anterior pituitary gland [5, 6].

The treatment often involved several hormone replacement therapies based on different kind of deficit. The prompt diagnosis and hormone replacement are essential for patient’s life quality and prognosis [7–9]. The clinical features at first appearance may include short stature, cryptorchidism, micropenis, delayed puberty, hypoglycemia or hypothyroidism. Considering the variety of clinical manifestations caused by hormone deficit, we hypothesize that the physical fitness is susceptible to unique body composition, muscular fitness or cardiopulmonary system, which yet no current studies have addressed with. Of an 18-year-old adolescent with exercise intolerance who is acquired with PSIS and had received growth hormone replacement therapy, we hereby present the result of the cardiopulmonary exercise test (CPET) which provides significant value in diagnosis, prognosis, and more importantly the exercise recommendation.

### Case presentation

**Fig. 1:**
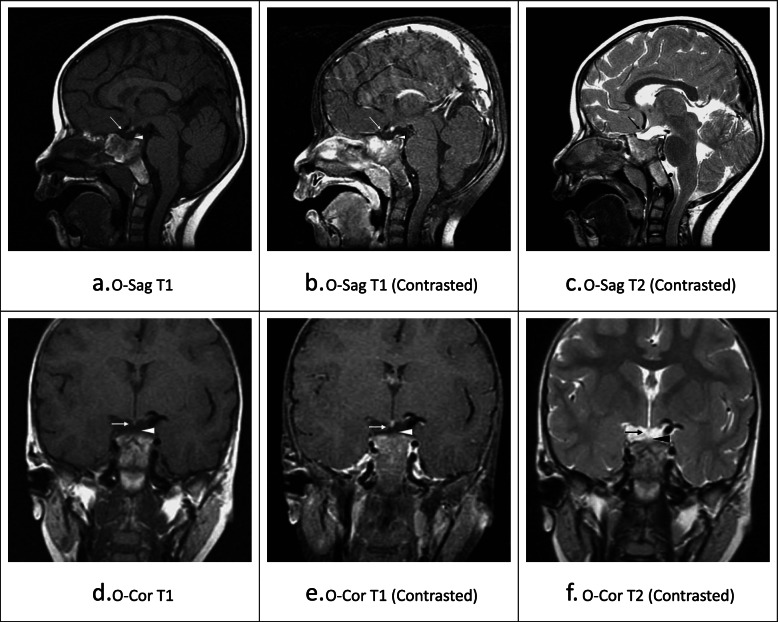
The sella magnetic resonance imaging (MRI) of the patient. The MRI (**a-f**) showed atrophic anterior pituitary lobe (arrow), disruption of pituitary stalk and ectopic posterior pituitary lobe (arrowhead) at the junction of the pituitary stalk and hypothalamus. No evidence of abnormal signal intensity mass lesion in the sella and suprasellar region was noted. The MRI scan series was labeled below each image. O-Sag = orthogonal sagittal; O-Cor = orthogonal coronal

A 3-year-and-5-month old child was first brought to our pediatric department due to failure to thrive with height 77.5 cm and body weight 6.2 kg, which were both below the 1th percentile on a growth chart. The father is 168 cm and the mother is 155 cm in height. Reviewing his gestational and birth history, the mother was first pregnant (gravidity: 1, parity: 1) and no serious complications were reported during the full-term pregnancy. The baby was born via spontaneous vaginal delivery with birth body weight 2760 gm. A series of initial evaluations and testing were conducted by having an impression of unspecified short stature. The brain magnetic resonance imaging (MRI) (Fig. 1) revealed atrophy of the pituitary gland and interruption of the pituitary stalk. Ectopic posterior pituitary stalk was also noted at the junction between the upper pituitary stalk and hypothalamus. The blood test showed decreased human growth hormone (< 0.15 ng/mL) yet with normal thyroid hormone level (HS-TSH: 1.2 uIU/ml, T3: 136 ng/dL, T4: 9.94 ug/dl). The insulin hypoglycemic test was arranged but postponed due to mild fever during the admission.

**Fig. 2:**
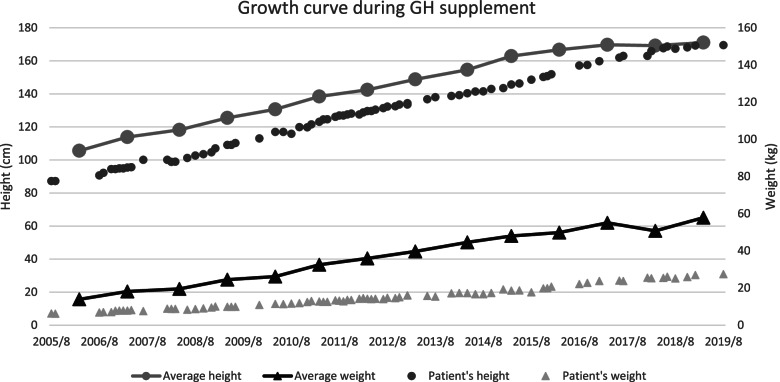
Growth curve during growth hormone supplement. The height and weight of the case were recorded throughout the therapy. The average height and weight of children and young adults in Taiwan (solid line) were obtained from Ministry of Health and Welfare, Taiwan, in which 2189 males of different ages in 2013 ~ 2016 were included [32]

After a few months, the patient was admitted again for thorough evaluations. The insulin hypoglycemic test showed a subnormal increase (0’: 0.14 ng/mL; 60’: < 0.1 ng/mL) in serum growth hormone with blood sugar 40 mg/dL after insulin injection (0.1 U/kg) pushed intravenously, indicating growth hormone (GH) deficiency. The morning cortisol level was 14.51 ug/dl, with HS-TSH: 1.873 uIU/ml, T3: 101 ng/dL and T4: 7.22 ug/dl, thus the patient was diagnosed with no adrenal insufficiency or hypothyroidism. Somatropin supplement therapy (0.025 ~ 0.035 mg/kg/day) was initiated at the age of 4 years and 5 months and discontinued at the age of 17 years and 2 months, when the hand bone age reached 17 years old. The growth curve is presented as (Fig. 2). At the age of 4 years and 5 months, the patient’s height was 80.6 cm with weight of 6.8 kg, and at the age of 17 years old his height was 150.7 cm with weight of 20.4 kg. The physical examination and blood test had been regularly repeated since progressive impairment of the residual pituitary function throughout childhood was reported in previous literature [10, 11]. At the age of 18 years old and 3 months, his testes were measured with orchidometer as 15 ml bilaterally in volume and stretched penis 8 cm in length, which were both considered within normal range. The blood test revealed low level of insulin-like growth factor 1 (IGF-1), but normal level of thyroid hormone, testosterone and other biochemical result (Table 1).

**Table 1:** Laboratory data at the age of 18 years and 3 months’ old

Free T4	1.3 ng/dL	(0.7—1.8)
i-PTH	34.9 pg/mL	(12 – 88)
Testosterone	9.21 ng/mL	(2.4–8.7)
Creatinine	1.00 mg/dL	(0.7–1.3)
Calcium	9.7 mg/dL	(8.6–10.3)
Phosphate	3.8 mg/dL	(2.5–5.0)
GPT	28 U/L	(0–40)
Alkaline-P	122 U/L	(34–104)
Albumin	5.1 g/dL	(3.5–5.7)
WBC	4240 /uL	(4100–10,500)
Segmented WBC	50.8%	(41.8–70.8)
Lymphocyte	38.2%	(20.7–49.2)
Hgb	16.1 g/dL	(13.4–17.2)
MCV	88.8 fL	(83.4–98.5)
Platelet	263,000 /uL	(160–370 × 10^3^)
IGF-1	64.7 ng/mL	(233.4—573.4)*

The IGF-1 level was significantly below the lower limit of the reference range with the same age and sex according to a population-based study with 2791 Chinese people enrolled [33]

*IGF-1* Insulin growth factor 1, *T4* Thyroxine, *i-PTH* Intact parathyroid hormone, *GPT* Glutamate pyruvate transaminase, *WBC* White blood cell, *Hgb* Hemoglobin, *MCV* Mean corpuscular volume

Considering the rare clinical condition along with exercise intolerance, the patient was referred to rehabilitation department for evaluations of physical fitness and exercise recommendations. Pulmonary function test (PFT) and CPET were arranged with informed consent and with no contraindications, in accordance with the recommendations of American College of Sports Medicine’s Guidelines for Exercise Testing and Prescriptions (ACSM guidelines), 10^th^ edition. [12]

The PFT was performed with a spirometry and data were collected including forced vital capacity (FVC), forced expiratory volume in one second (FEV1), and maximal voluntary ventilation (MVV). The settings of CPET composed of a treadmill, a flow module, a gas analyzer, and an electrocardiographic monitor. A detailed demonstration was given before the test. The patient was in his regular health status and could understand and follow the instructions. The symptom-limited exercise testing with ramped Bruce protocol was then conducted. Collected parameters included heart rate (HR), blood pressure (BP), minute ventilation (VE), oxygen uptake (VO_2_), carbon dioxide output (VCO_2_), respiratory exchange ratio (RER) and partial pressure of end-tidal carbon dioxide (PETCO_2_). The predicted maximal heart rate (HRmax) was calculated as 201 using the Eq. 216.6 – (0.84 × Age) [13]. The VO_2_ (ml/kg/min) was recorded breath-by-breath during the testing and divided by 3.5 to present exercise capacity as metabolic equivalent of tasks (METs). The predicted maximal oxygen uptake (VO_2_max pred.) was determined by the age, gender and weight [14]. HR recovery (HRR) was defined as the difference between HR at 1 min after testing and peak HR. The anaerobic threshold (AT) was determined by VE/VO_2_ and VE/VCO_2_ methods.

The maximal oxygen uptake (VO_2_max) was determined if any of the following criteria had ever met: 1.) A plateau in VO_2_ with increased workload, 2.) Failure of HR to increase with increases in workload, 3.) A peak RER ≥ 1.10. The test was terminated as the patient demanded for stop due to severe fatigue and leg soreness. The maximal effort was considered attained as peak RER reached over 1.10. During the testing, there was no angina, cyanosis or dizziness, and no ST elevation or displacement noted on electrocardiographic monitor. The heart rate and blood pressure increased steadily with incremental work load. The baseline measurements and the results were illustrated in (Table 2) and (Table 3).

**Table 2:** Baseline characteristics before cardiopulmonary exercise test

Weight (kg)	26.3
BMI (kg/m^2^)	11.3
Resting SBP (mmHg)	100
Resting DBP (mmHg)	65
Resting HR (bpm)	74
FVC (L)	2.2
FVC, % of predicted	80.7%
FEV1 (L)	1.89
FEV1, % of predicted	81.2%
FEV1/FVC	85.9%
MVV (L)	48.75
PETCO_2_ (L)	35
Height (cm)	151.3

*BMI* Body mass index, *SBP* Systolic blood pressure, *DBP* Diastolic blood pressure, *HR* Heart rate, *FVC* Forced vital capacity, *FEV1* Forced expiratory volume in one second, *MVV* Maximal voluntary ventilation, *PETCO*_*2*_ End‐tidal carbon dioxide

**Table 3:** The results of cardiopulmonary exercise test

OUES	1.0
VE/VCO_2_ slope	26.5
VO_2_/WR slope (mL/min/watt)	6.9
*Anaerobic threshold*	
AT HR (bpm)	149
AT VO_2_ (mL/min)	23.1
AT MET	6.6
AT VE (L)	17.0
AT RER	0.92
AT PETCO_2_ (L)	41
*Peak exercise*	
Peak HR (bpm)	174
Peak HR, % of predicted	86.6%
Peak SBP (mmHg)	136
Peak DBP (mmHg)	61
Peak VO_2_ (L/min)	0.82
Peak MET	8.9
**Peak MET, % of predicted**	**54%**
Peak VE (L)	26.9
BR, % of MVV	44.82%
Peak RER	1.14
Peak PETCO_2_ (L)	43
HRR (beats)	10

*HRR* Heart rate recovery, *OUES* Oxygen uptake efficiency slope, *VO*_*2*_ Oxygen consumption, *WR* Work rate, *PETCO*_*2*_ End‐tidal carbon dioxide, *MET* Metabolic equivalent of task, *VE* Minute ventilation, *BR* Breathing reserve, *RER* Respiratory exchange ratio

The peak heart rate was 86.6% of predicted HRmax in the presence of maximal effort indicating chronotropic incompetence less likely in this patient. The heart rate reserve (HRR) was 10 and much lower than 27, the mean value in 17–18-year-old healthy boy subjects reported by Singh et al. [15] which provided indirect evidence of autonomic imbalance. The maximal exercise capacity was 8.9 METs (VO_2_max: 31.15 ml/kg/min) and 54% of predicted value, showing moderate functional aerobic impairment. The breathing reserve was 44.82% and not characteristic of pulmonary diseases (< 20%). [16] The VE/VCO_2_ slope was 26.5 suggesting sufficient ventilatory efficiency. Above all, as the result hardly met the criteria for cardiovascular or pulmonary limitation, the clinical exercise intolerance could attribute to physical unfitness including low muscle mass and endurance.

According to the CPET result, the patient should face no obstacles to perform activities of daily livings, to carry out household work (ex: washing cars, mowing lawn), to participate in leisure time or sports (ex: badminton, golfing, table tennis) but to be cautious in some of severely vigorous activities (ex: competitive soccer game, carrying heavy bricks). [17] To improve his physical fitness and maintain health, a well-designed exercise prescription is suggested based on ACSM’s recommendation, which could employ the ‘’FITT-VP’’ principle including frequency, intensity, time, type, volume and progression. We hereby suggest an aerobic exercise program which is at least 30 min per day, 5 days per week with moderate intensity. The intensity is calculated with oxygen uptake reserve method [18]. The target VO_2_ (ml/kg/min) equals (31.15—3.5) x (40 ~ 60%) + 3.5, which is 14.56 ~ 20.09 ml/kg/min, to be more specific, jogging on even ground at rate of 3.3 ~ 6.6 km/hr. [12] The volume is about 750 MET·min/week and the exercise time is expected to increase 5 min every 2 week in the first month.

In addition, balanced nutrition intake is as well important and resistance training program could be helpful, especially in our case suspected of low muscle mass. Barbieri et al. has reported several benefits can be achieved through strength training in adolescents and children, including improved motor skills, body composition, reduced fat mass and bone health, [19] and accordingly we recommend a resistance exercise program which is 2 days per week, 3 sets of 10 repetitions involving major muscle group with light intensity (50% 1-RM). The interval between each session should be at least 2 days. [12] The multi-joint exercises (such as chest press, squats) are preferred in avoidance of muscle imbalances or even injury. As the patient never participates in resistance training, it is important to select reasonable weight during training and gradually increase the volume after the patient is familiar with the proper technique. Even as few as one set per muscle group per session can result with significant strength gains. [20]

## Discussion

In the present study, we described long-term follow up of clinical and hormonal characteristics of an 18-year-old adolescent diagnosed as PSIS and the exercise capacity after GH supplement therapy with objective evaluations by CPET. We analyzed the test result to specify the main cause limiting exercise performance and furthermore, to provide detailed exercise prescription for better clinical outcome.

PSIS is associated with different level of anterior pituitary insufficiency while the posterior pituitary function often remains intact. Patients are mostly referred for growth retardation, as also reported are neonatal hypoglycemia or malformation. [21] In our case, the patient was diagnosed with PSIS based on endocrine study and MRI findings. Isolated GH deficiency has been noted and managed since youth. The total height gain standard deviation score (SDS) was 1.39, though short of target adult height (160 ± 6.5 cm), which is in accordance with previous studies (mean height gain SDS: 2.3; 0.7 ~ 5.4). [11, 22] The hormone impairment level of PSIS could range from isolated GH deficiency to combined pituitary hormone deficiency, typically progressing gradually to permanent pan-hypopituitarism in adulthood. Our patient developed mature second sex characteristics (Tanner stage IV) with normal level of gonadotropic steroids and other hormones. Considering the limited present studies and the high heterogeneity of the diseases, we perceived our case at less severe end of the broad spectrum. No hormone supplement is currently indicated in our case but regular check-up is suggested.

The exercise capacity of our case is lower than predicted and it is consistent with previous studies, but the effect of GH replacement on the exercise capacity remains equivocal. Whitehead et al. and Nass et al. demonstrated the maximal oxygen uptake of patients with acquired growth hormone deficiency (AGHD) is significantly lower than normal sex- and age-matched individuals. [23, 24] They also reported that GH replacement improves exercise capacity significantly. On the other hand, a double-blind, placebo-controlled randomized, cross-over trial enrolled 17 patients with acquired hormone deficiency to receive recombinant human growth hormone or placebo, but the exercise capacity remained unchanged. [25] Another similar study suggested that 3 months of GH replacement improved muscle oxidative capacity by comparing succinate dehydrogenase density in quadriceps muscle biopsies, which should reflect an improvement in exercise capacity. [26] However, no significant effects in peak VO_2_, VE/VCO_2_ slope, anaerobic threshold RER, exercise time were noticeable, while the authors considered it could be contributed by short period of therapy, low dose of GH or an obese population. Of note, most of the studies focused on acquired GH deficiency instead of primary GH deficiency because of the small research population and associated studies should be conducted in the future.

No available studies analyzing CPET result of PSIS patients were submitted before the present study. A thorough integrative interpretation of multiple CPET parameters prompts a proper differential diagnosis. According to the Fick’s equation, oxygen uptake is the product of the cardiac output and the arteriovenous difference, which respectively reflecting central oxygen supply and peripheral oxygen demand. [27] The decreased exercise captivity could possibly be contributed by pulmonary, cardiovascular and metabolic diseases or physical unfitness. The heart rate reserve (HRR) is considered to reflect the balance of the autonomic nervous system with the initial fall activated through parasympathetic system and followed by sympathetic system withdrawal. [28] Lower value in HRR is indicative of increased cardiovascular risk, metabolic risk and worse exercise capacity, which may be improved with regular physical activity participation. [15, 29] A high VE/VCO_2_ slope can be observed in patients with heart failure, pulmonary hypertension, chronic obstructive pulmonary disease or restrictive pulmonary disease. [16] Chua et al. has shown a VE/VCO_2_ slope > 34 a higher risks for death and hospitalization due to decompensated chronic heart failure [30], while Ritt et al. suggested the best cutoff point for worse prognosis was 32.5. [31]

There are limitations in the present study. First, even though PSIS pathogenesis and etiology has not been fully understood, there have been great interests and improvements in the field of genetic studies, but the genetic surveillance was not conducted as the family could not afford the price. Secondly, the objective evaluations for body composition such as bioelectrical impedance analysis (BIA) or dual energy X-ray absorptiometry (DEXA) is lacking, which could provide more direct evidence of suggested low muscle mass. Third, additional CPET is warranted after aerobic and resistance exercise training to better evaluate the effect on physical fitness, which is unable to be presented here while the rehabilitation program has not completed. Nevertheless, the drawbacks would not affect the testing result and subsequent differential diagnosis. Above all, regular follow up for disease progression as well as effects of exercise is warranted.

## Conclusion

To the best of our knowledge, this is the first study conducting CPET to evaluate the physical fitness of PSIS patient. The testing result provided evidence of moderate functional impairment (54% of predicted VO_2_max) mainly affected by physical unfitness without significant cardiovascular or pulmonary limitations. CPET serves as a valuable tool for diagnostic purpose. Aerobic and resistance exercise training for the patient should be conducted promptly for better prognosis but under safe circumstances, with criteria which could be provided by CPET results.

## Data Availability

The datasets used and/or analyzed during the current study are available from the corresponding author on reasonable request.

## Abbreviations

1-RM: One-repetition maximum
AT: Anerobic threshold
BIA: Bioelectrical impedance analysis
BP: Blood pressure
CPET: Cardiopulmonary exercise test
DEXA: Dual energy X-ray absorptiometry
FEV1: Forced expiratory volume in one second
FVC: Forced vital capacity
GH: Growth hormone
HR: Heart rate
HRmax: Maximal heart rate
HRR: Heart rate recovery
IGF-1: Insulin-like growth factor 1
METs: Metabolic equivalent of tasks
MRI: Magnetic resonance imaging
MVV: Maximal voluntary ventilation
PETCO2: Partial end-tidal carbon dioxide
PSIS: Pituitary stalk interruption syndrome
RER: Respiratory exchange ratio
VCO2: Carbon dioxide output
VE: Minute ventilation
VO2: Oxygen uptake
VO2max: Maximal oxygen uptake
VO2max pred: Predicted maximal oxygen uptake
